# Cross-feeding interactions between human gut commensals belonging to the *Bacteroides* and *Bifidobacterium* genera when grown on dietary glycans

**DOI:** 10.20517/mrr.2021.05

**Published:** 2022-03-18

**Authors:** Pedro Fernandez-Julia, Daniel M. Commane, Douwe van Sinderen, Jose Munoz-Munoz

**Affiliations:** ^1^Microbial Enzymology Group, Department of Applied Sciences, Ellison Building A, University of Northumbria, Newcastle Upon Tyne NE1 8ST, UK.; ^2^APC Microbiome Ireland and School of Microbiology, University College Cork, Western Road, Cork T12 YT20, Ireland.

**Keywords:** Cross-feeding, plant fibre, *Bacteroides*, *Bifidobacterium*, gut microbiota, prebiotic

## Abstract

Elements of the human gut microbiota metabolise many host- and diet-derived, non-digestible carbohydrates (NDCs). Intestinal fermentation of NDCs salvages energy and resources for the host and generates beneficial metabolites, such as short chain fatty acids, which contribute to host health. The development of functional NDCs that support the growth and/or metabolic activity of specific beneficial gut bacteria, is desirable, but dependent on an in-depth understanding of the pathways of carbohydrate fermentation. The purpose of this review is to provide an appraisal of what is known about the roles of, and interactions between, *Bacteroides *and *Bifidobacterium* as key members involved in NDC utilisation. *Bacteroides* is considered an important primary degrader of complex NDCs, thereby generating oligosaccharides, which in turn can be fermented by secondary degraders*.* In this review, we will therefore focus on *Bacteroides* as an NDC-degrading specialist and *Bifidobacterium* as an important and purported probiotic representative of secondary degraders. We will describe cross-feeding interactions between members of these two genera. We note that there are limited studies exploring the interactions between *Bacteroides *and* Bifidobacterium*, specifically concerning β-glucan and arabinoxylan metabolism. This review therefore summarises the roles of these organisms in the breakdown of dietary fibre and the molecular mechanisms and interactions involved. Finally, it also highlights the need for further research into the phenomenon of cross-feeding between these organisms for an improved understanding of these cross-feeding mechanisms to guide the rational development of prebiotics to support host health or to prevent or combat disease associated with microbial dysbiosis.

## INTRODUCTION

**Figure 1:**
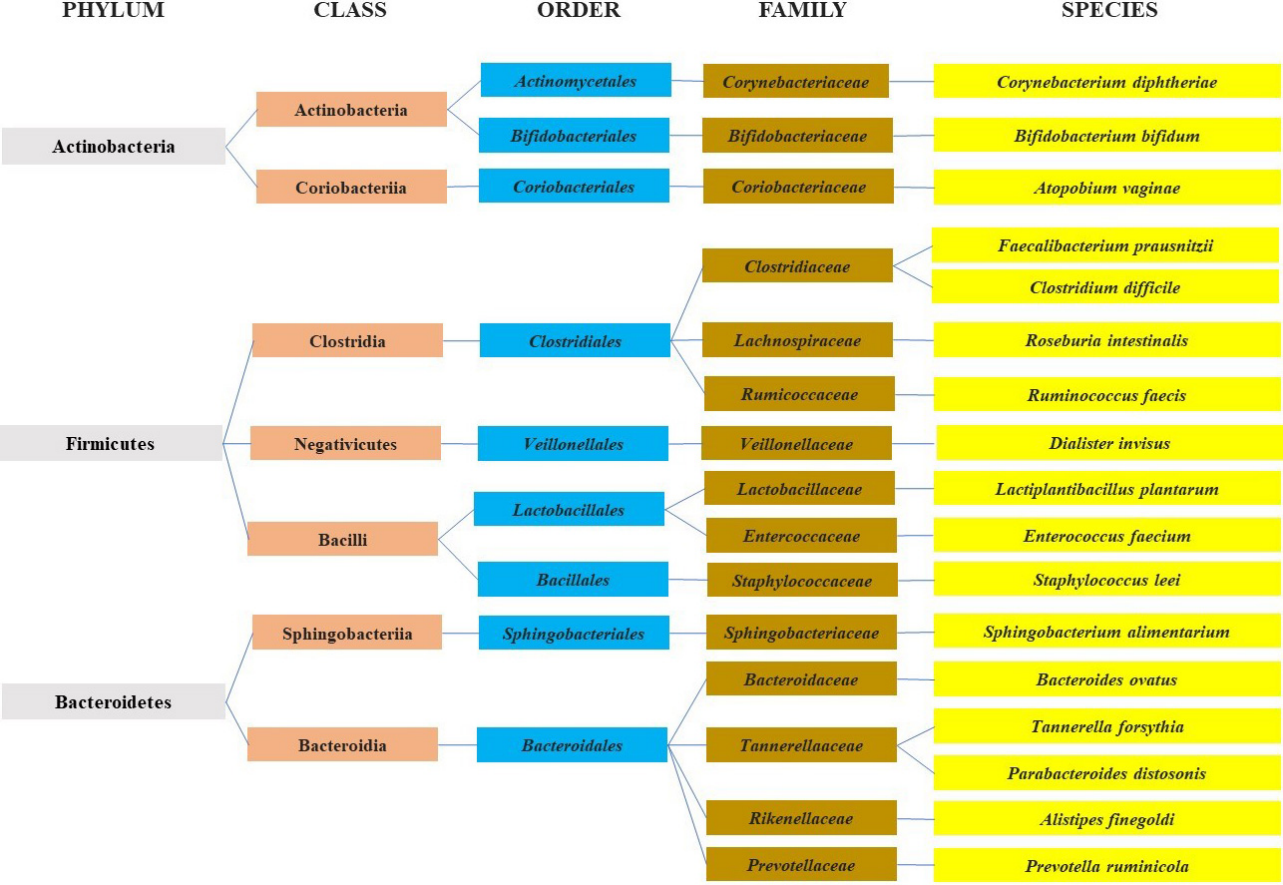
A diagram representing the most abundant microbial components of the human microbiota in the colonic section of the gut.

The diverse community of microorganisms that inhabit the human gastrointestinal tract make up the human gut microbiota (HGM)^[1]^. The HGM consists of protozoa, archaea, eukaryotes, viruses and bacteria, and these organisms have evolved to exist symbiotically within the host, exerting various beneficial roles, including but not limited to, energy retrieval, protection from invading pathogens, maintaining gut homeostasis, and modulating the immune system. The HGM is made up of three main phyla: Firmicutes, Bacteroidetes and Actinobacteria [Figure 1]^[2]^.

Changes in the composition of the gut microbiota can be influenced by various factors, such as type of diet, stress or environment, and may result in a so-called state of dysbiosis, an imbalance in the levels of members of the HGM that has been linked to various diseases. Depletion or overabundance of certain bacterial species throughout (part of) an individual’s lifetime may contribute to gut disorders such as inflammatory bowel disease, which includes Crohn’s disease and ulcerative colitis^[3]^. In this sense, it has been shown that a gut microbiota imbalance during infancy can provoke the development of diseases (e.g., auto-immune diseases) at a later life stage^[4]^. Moreover, metabolic products of the gut microbiota may promote diseases such as colorectal cancer and obesity and can affect signalling pathways within the host.

The complex structural variation of dietary polysaccharides, such as arabinoxylan, arabinogalactan or pectin, is determined by the monosaccharide composition, type of glycosidic linkage, side chains and substitutions^[5]^. Due to the high degree of structural diversity found among non-digestible carbohydrates (NDCs) that are part of the human diet, efficient metabolism of dietary and host-derived polysaccharides requires an array of highly specific enzymes, that all belong to the so-called Carbohydrate active enzymes (CAZymes)^[6]^. Host-derived polysaccharides such as mucins or glycosaminoglycans, and diet-derived polysaccharides, which include glucans, xylans, fructans, mannans and galactans, are mostly resistant to degradation by human enzymes and therefore they form fermentable substrates for members of the gut microbiota, especially in the colon^[7]^. Fermentation of dietary fibre by the gut microbiota typically produces short-chain fatty acids (SCFAs), such as acetate, propionate and butyrate, which are taken up by the host and used primarily as an energy source, thereby benefitting host health^[8,9]^. In addition, *Bifidobacterium* species produce lactate, as a major metabolic end-product, while some species generate 1,2-propanediol from fucose metabolism, and both of these metabolites can in turn be converted into SCFAs by other gut microbiota members. These metabolites affect various biological processes; in the intestinal lumen SCFAs lower the intestinal pH, which may inhibit the growth of pathogens, while this is also believed to influence the composition of the wider microbial community^[10]^. In intestinal epithelial cells, butyrate regulates cell proliferation, differentiation and gene expression in a manner associated with antineoplastic transformation. Systemically, propionate and acetate activate the free fatty acid receptors GPR41 and GPR43, which are expressed by enteroendocrine cells lining the gut and are present in other tissues, including adipocytes; these receptors may help to activate anti-inflammatory signalling pathways, control metabolism, and influence appetite and mood^[11]^. The ability of the resident bacteria in the gut to efficiently metabolize these otherwise indigestible glycans means that they share a symbiotic relationship with the host and with one another as these bacteria must effectively compete for carbohydrate nutrition and so have co-evolved efficient glycan-harvesting strategies.

**Figure 2:**
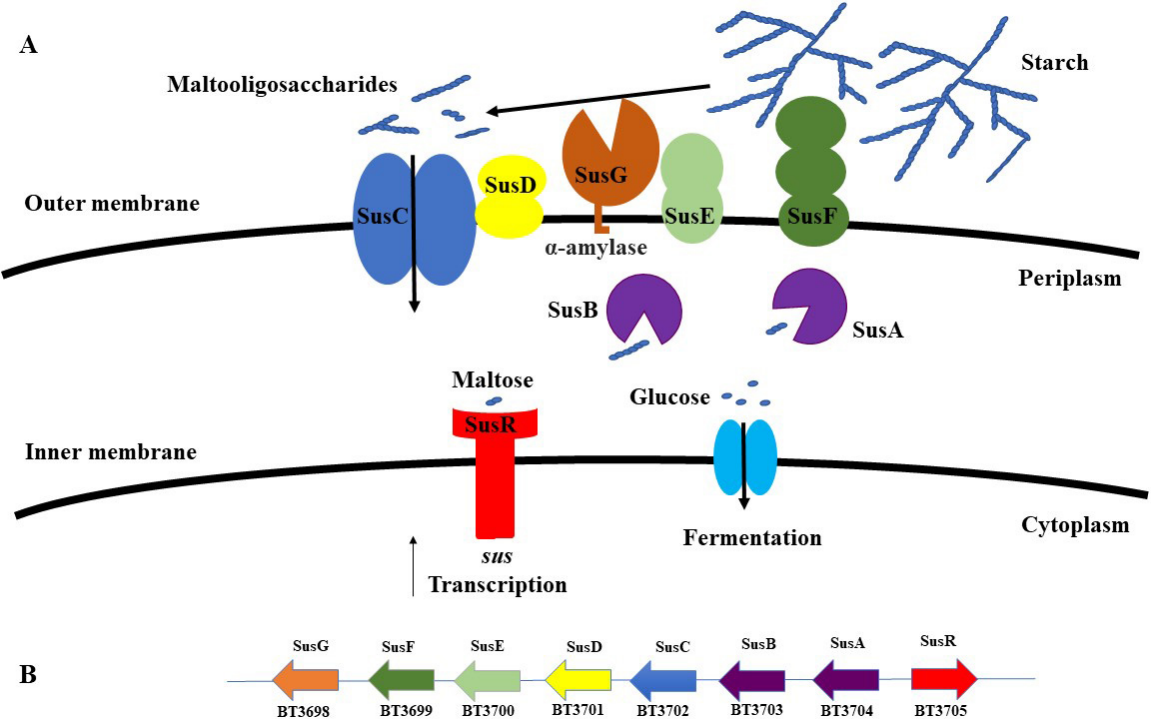
(A) A schematic overview of the PUL required for starch utilisation (Sus) in* B. thetaiotaomicron *VPI-5182*. *The substrate is bound by lipoproteins at the cell surface, where it is initially degraded to oligosaccharides and then transported to the periplasm, where it is further hydrolysed to monosaccharides which can then be transported into the cytoplasm of the cell. (B) Genomic content of *B. thetaiotaomicron *VPI-5182 PUL in its action on starch. PUL: Polysaccharide utilisation loci.

The Gram-negative Bacteroidetes is one of the main phyla that make up the HGM. A large proportion of the genome of these organisms is dedicated to the metabolism of complex glycans, making them primary glycan degraders in the HGM^[12]^. Although several *Bacteroides* species are known to degrade a variety of distinct polysaccharides, there is not a single known species capable of metabolising all main dietary, complex glycans, such as starch, pectin, xylan or beta-glucan. The degradation of such complex carbohydrates is performed by CAZymes, whose encoding genes are arranged into clusters known as polysaccharide utilisation loci (PULs). These PULs encode, in addition to the above mentioned CAZymes [represented by glycoside hydrolases (GHs), polysaccharide lyases and carbohydrate esterases], various other proteins involved in glycan degradation, including cell surface glycan-binding proteins, TonB-dependent transporters, and sensors, which regulate PUL transcription^[13]^. For example, the first described PUL was the starch utilisation system (Sus-system) of *Bacteroides thetaiotaomicron *VPI-5182, which has been extensively studied. The cell surface-associated proteins SusE and SusF bind the polysaccharide substrate, thereby facilitating the initial starch degradation by SusG - an amylase enzyme, which is also located at the bacterial cell surface, and which hydrolyses the large polymeric substrate into smaller oligosaccharides. These oligosaccharides are then transported into the periplasmic compartment by the SusC/SusD complex (a TonB-dependent transporter), where they are further degraded into monosaccharides by SusA and SusB glycosidases [Figure 2].

Firmicutes and Actinobacteria are the other two main phyla that are resident in the human gut. These phyla represent Gram-positive bacteria that possess different mechanisms for glycan degradation^[14]^. Gram-positive PULs (gpPULs) have a similar gene content to the PUL systems present in Bacteroidetes. However, gpPULs are devoid of the SusC/D-encoding gene pair that characterises an archetypical PUL^[15]^. The gpPULs encode proteins such as transporters (commonly ABC-type transporters), regulatory proteins (typically LacI-type regulators) and glycan-degrading enzymes (mostly GHs), and large glycans (i.e., those that have a DP of > 6-7 monosaccharidic moieties) must be degraded by extracellular (or cell envelope-bound) glycoside hydrolases. The resulting oligosaccharides are then typically bound by solute binding proteins associated with an ABC uptake system which internalizes them to be further degraded into monosaccharides.

In the HGM, the phylum Actinobacteria is largely represented by the *Bifidobacterium* genus. Members of this genus mainly produce lactate, acetate and formate (and sometimes 1,2-propanediol) as products of sugar fermentation in the gut^[16]^. Analysis of the genomes of *Bifidobacterium longum* subspecies *infantis *and *Bifidobacterium bifidum* show that they encode glycosyl hydrolases that are utilised in the degradation of human milk oligosaccharides^[17]^. *Bifidobacterium* species, however, typically degrade glycans with a lower degree of polymerisation than those utilized by Bacteroidetes members.

As mentioned above, *Bifidobacterium* species are generally able to metabolize a range of oligomeric and rather simple carbohydrates, commonly being unable to metabolise more complex polysaccharides that require extracellular GHs. Studies have reported evidence of cross-feeding behaviour between members of the *Bifidobacterium* genus with other organisms, thereby allowing the complete metabolism of certain complex glycans^[18]^. An example of this is the interspecies cross-feeding relationship between *Bifidobacterium bifidum *and *Bifidobacterium breve* when co-cultured on sialyllactose^[19]^. This study showed how *Bi. breve *was able to cross feed on sialic acid, the product of 3’-sialyllactose degradation by *Bi. bifidum*. *Bifidobacterium* species have also been shown to produce metabolites, such as 1,2-propanediol, that can be used by other HGM members, e.g., *Lactobacillus spp.* or *Eubacterium hallii*, for cross-feeding purposes^[20,21]^.

This review will focus on the molecular mechanisms underpinning cross-feeding interactions between members of *Bacteroides* and* Bifidobacterium* pertinent to the metabolism of dietary glycans, such as arabinoxylans. We selected these two genera because they are extensively studied as primary and secondary degraders of these glycans. Members of the genus *Bacteroides* have been shown to be glycan generalists that metabolize complex polysaccharides, generating extracellular oligosaccharides, which may be shared with other microbial members in the gut, such as particular *Bifidobacterium* species. The latter bacteria have been shown to generally act as specialists in the metabolism of oligosaccharides rather than complex intact polysaccharides. In addition, as we stated above, bifidobacteria are considered to represent beneficial microbes supporting human health. Arabinoxylans are present in commonly consumed cereals, like wheat or corn, and the cross-feeding behaviour between these two groups of bacteria demonstrates how they have co-evolved to utilise a varied range of diet and host-derived carbohydrate sources. We will explore both intra- and inter-genus interactions among members of these two genera. Although other metabolites have been shown to be involved in the cooperation or inhibition of different members of gut microbiota, such as 1,2-propanediol or methane, this review will focus on carbohydrates and their metabolic end products.

## MICROBIAL INTERACTIONS FOR THE UTILISATION OF POLYSACCHARIDES

Microbial communities have evolved a balanced and dynamic network of metabolic interactions, which enable them to adapt and thrive within the human gastrointestinal tract (GIT). The competition for nutrients between members of the HGM has resulted in the evolution of ecological feeding strategies that increase the efficiency of glycan utilisation^[22]^. Although there are various distinct definitions describing such interactions in literature, Smith *et al.*^[22]^ have defined bacterial metabolic cross-feeding as “an interaction between bacterial strains in which molecules resulting from the metabolism of one strain are further metabolised by another strain”. The term “microbial syntrophy” is a closely related term to cross-feeding, which describes the obligately mutualistic metabolism of microorganisms whereby processes are carried out through metabolic interactions between organisms that are mutually dependent upon one another^[23]^. These syntrophic interactions can result in the metabolism of complex molecules, which would otherwise be unable to be degraded by the action of just one organism.

### *Bacteroides*-*Bacteroides* interactions

The ability of various* Bacteroides* species to utilise polysaccharides has been well documented^[24]^. Initial *Bacteroides* processing of complex NDCs typically results in the extracellular release of polysaccharide breakdown products (PBPs) which then become available to other organisms that are unable to directly metabolise the complex NDC. Rakoff-Nahoum *et al.*^[25]^ proposed this notion in 2014 and found that various *Bacteroides* species are able to degrade different plant-derived polysaccharides to varying degrees, thus producing varying amounts of PBPs. Interestingly, they observed that *B. ovatus *ATCC 8483 and *B. vulgatus *ATCC 8482 released only oligosaccharide PBPs from xylan breakdown, which contradicts the findings of Salyers *et al.*^[26]^ in 1981, who previously described the production of xylose on the breakdown of xylan by these organisms. They then performed growth experiments showing that these PBPs were not universally utilised by the non-polysaccharide-utilising organisms meaning that the use is dependent upon the producer strain as well as the PBPs produced. The ability of the non-utilising strains to grow on these PBPs suggests that they possess PULs that encode CAZymes required to metabolise the breakdown products. The observation of amylopectin breakdown in extracellular zones suggests that, as a consequence of releasing PBPs by the utilising strains, GH and PL CAZymes are also secreted. Communication between Gram-negative bacteria via outer membrane vesicles (OMVs) allows for the secretion of enzymes to extracellular regions where polysaccharide degradation can take place. This was demonstrated in^[26]^ using western immunoblot analysis, which revealed that GH and PL enzymes were present in OMVs from *B. ovatus *ATCC 8483 and that their release supported the growth of *B. vulgatus *ATCC 8482 on inulin. This evidence is supported by a further study by Rakoff-Nahoum *et al.*^[27]^ in 2016, which found that there is a co-operation between *B. ovatus *ATCC 8483, as primary degrader, in the breakdown of inulin releasing two GH enzymes (BACOVA_04502 and BACOVA_04503) that are not necessary for its metabolism. It is thought that *B. ovatus* ATCC 8483 expresses these enzymes for the purpose of feeding other organisms in its microbial community and that this process was beneficial for *B. vulgatus *ATCC 8482, which cannot digest inulin. In co-culture, *B. vulgatus *ATCC 8482 was shown to grow with increased fitness and in return, increased the fitness of *B. ovatus *ATCC 8483 possibly due to the production of molecules, PBP or other metabolites, that support the growth of *B. ovatus *ATCC 8483 or by the detoxification of substances that inhibit its growth. These studies highlight the interactions between members of the *Bacteroides* genus that allow the growth of organisms that do not possess the necessary degradative enzymes for the direct utilisation of certain carbohydrates. These interactions may be key in the establishment of a metabolically dynamic functional community of microorganisms in the human GIT.

### *Bifidobacterium*-*Bifidobacterium* interactions

*Bifidobacterium* species have also been known to interact with one another to cooperatively break down carbohydrates^[28]^. The differing abilities of strains to utilise glycans have been thought to result in the evolution of cross-feeding activities between bifidobacterial strains. Studies have shown that *Bifidobacterium *species are able to secrete GH enzymes^[29]^. It is predicted that 10.9% of the GH enzymes encoded by *Bifidobacterium* are extracellular, of which 24% are predicted to be of the GH43 family, which act as β-xylosidases and α-L-arabinofuranosidases. Extracellular GH enzymes were identified in 43 bifidobacterial species, with the most prevalent being *Bifidobacterium biavatii* which was identified to secrete 17 GHs, while *Bifidobacterium scardovii *and *Bi. bifidum *each secreting 11 GHs^[29]^. This provides a strong indication for the existence of glycan sharing abilities among bifidobacterial strains. For example, co-cultivation of *Bi. bifidum* PRL2010 with *Bi. breve* 12L, *Bifidobacterium adolescentis* 22L and *Bifidobacterium thermophilum* JCM1207 supports the growth of *Bi. bifidum* PRL2010^[28]^. Furthermore, metabolic activity of *Bi. bifidum* PRL2010 was enhanced when co-cultivated with these other bifidobacterial strains, and transcription of genes involved in carbohydrate metabolism was enhanced. When grown in monoculture, *Bi. bifidum *PRL2010 was unable to utilise starch or xylan; however, the growth of PRL2010 was observed when this strain was cultivated together with *Bi. breve* 12L or *Bi. adolescentis* 22L. Conversely, a decrease in the growth of *Bi. breve* 12L and *Bi. thermophilum* JCM1207 was noted, suggesting that the utilisation of starch or xylan by *Bi. bifidum* PRL2010, respectively, did not benefit these strains and in fact imposed competitive pressure on these strains. The reduction in lactate and acetate production by* Bi. breve* 12L when grown in co-culture as opposed to monoculture supports the idea that the growth of this strain is hindered by the presence of another *Bifidobacterium *species. Transcriptomic analysis showed that the genes encoding an ABC-transporter and an MFS transporter in *Bi. bifidum *PRL2010 was upregulated when grown in co-culture. It was suggested that this upregulation was due to the production of simple carbohydrates by *Bi. breve* 12L and *Bi. adolescentis *22L, which act as PBPs that can be utilised by *Bi. bifidum* PRL2010. Interestingly, upregulation of 21 genes of *Bi. breve* 12L was observed when co-cultures with *Bi. bifidum* PRL2010 on starch, and 42 genes when grown on xylan. This upregulation provides evidence for a mutualistic relationship between these two strains^[28]^.

In addition to cross feeding on plant-derived glycans, certain members of the *Bifidobacterium* genus are involved in the metabolism of host-derived glycans and human milk oligosaccharides (HMOs)^[19,30]^ (Egan *et al.*^[30]^, 2014). *Bi. breve* UCC2003 is a known utiliser of sialic acid, which is a monosaccharide present in mucin and certain HMOs, and which is released by *Bi. Bifidum *PRL2010 when grown on such carbohydrate substrates due to the particular extracellular GH enzymes^[30,31]^. The production of sialic acid from the degradation of the HMO 3’ sialyllactose by *Bi. bifidum *PRL2010 was shown to support the growth of *Bi. breve* UCC2003^[19]^.

### *Bacteroides*-*Bifidobacterium* interactions

Research has shown that *Bacteroides* and *Bifidobacterium* have a cross-feeding relationship in the utilisation of certain dietary and host-derived carbohydrates^[18]^. The sharing of partially degraded oligosaccharides, intermediary molecules, and genes by lateral gene transfer, contributes to the metabolic flexibility of the HGM. In 2010, Hehemann *et al.*^[32]^, proposed that *Bacteroides plebeius*, a gut bacterium mainly present in the Japanese population*,* acquired genes from the marine bacterium *Zobellia galactanivorans* needed for the degradation of an algal polysaccharide, porphyran. This gene transfer was explained because seaweed is a common component of the Japanese diet, and bacteria associated with this nutrient may have been the route by which the novel CAZymes were acquired by *Bacteroides plebeius*. *Bacteroides* species often act as primary degraders of complex carbohydrates in the gut, releasing oligosaccharides that then become available for secondary degrader organisms^[33]^. Such cross-feeding activities are believed to allow less dominant organisms such as *Bifidobacterium* to utilise glycans as a source of nutrition without becoming completely out-competed by bacterial strains that are present in the gut in much higher numbers.

An investigation into the interactions between members of the* Bacteroides *and *Bifidobacterium* genera in the utilisation of different carbon sources was carried out by Rios-Covian *et al.*^[34]^. They cultured different combinations of two strains of *Bacteroides* and two strains of *Bifidobacterium* using exopolysaccharide, inulin or glucose as the sole carbon source. They found that *Bi. longum* NB677 and *Bi. breve* IPLA2004 are able to utilise glucose as a carbon source; however, neither grew on more complex polysaccharides. Conversely, *B. thetaiotaomicron *DSM 2079 and *B. fragilis* DSM 2151 were able to utilise all tested carbon sources. The authors found that the growth of *B. thetaiotaomicron* DSM 2079 on glucose was inhibited by the presence of *Bi. breve *IPLA2004, suggesting that the interaction between these organisms, when grown on this simple sugar, is not mutualistic. *B. fragilis* DSM 2151, on the other hand, was shown to increase the fitness of *Bi. longum* NB677. These results emphasized that the behaviour of co-cultures of different species is not universal and very much depends on the specific bacterial strain/species combination and particular carbon source.

**Figure 3:**
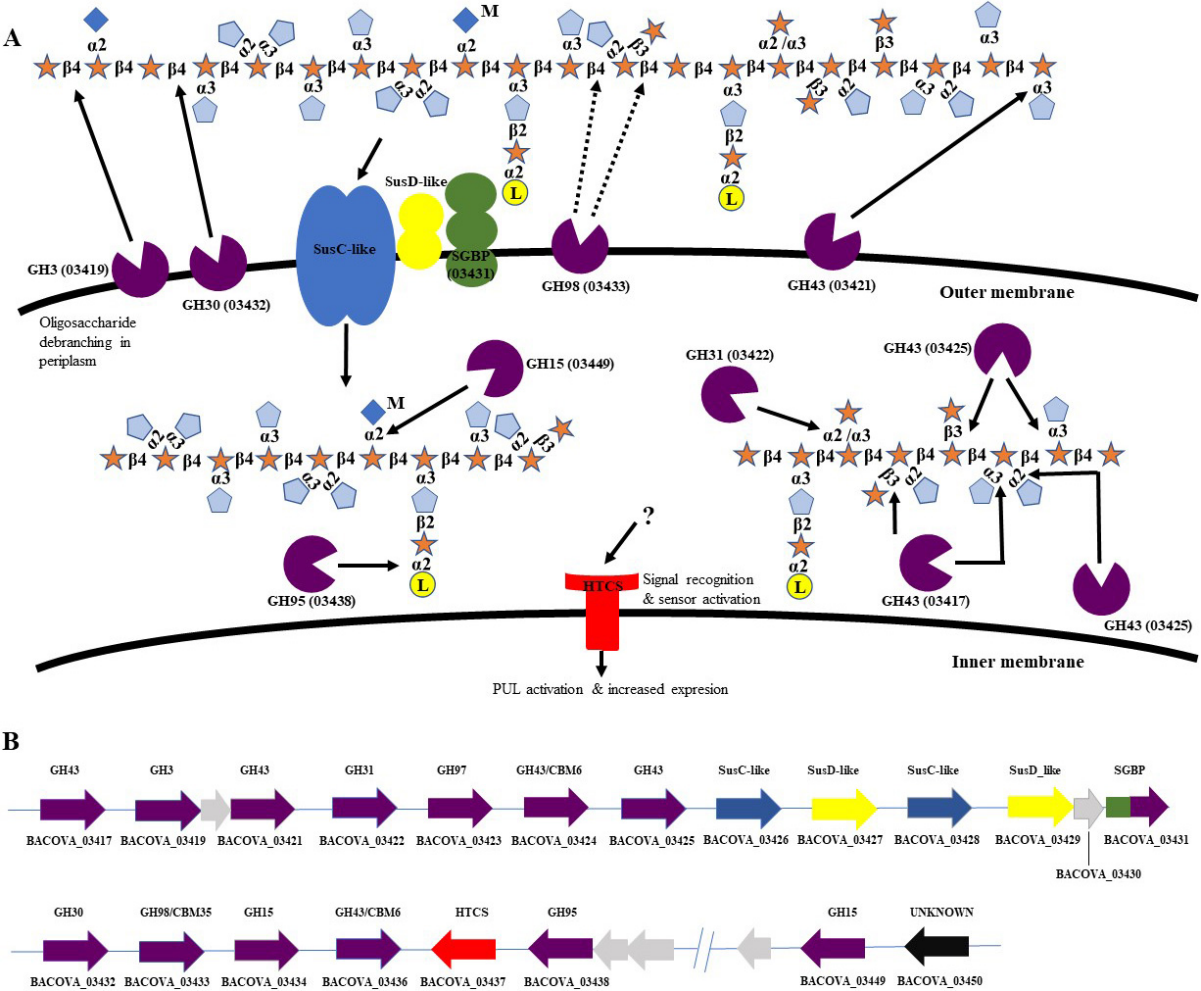
(A) A schematic representation of the glucoronoarabinoxylan utilisation system in *B. ovatus* ATCC 8483. The substrate is bound by lipoproteins at the cell surface where it is primarily degraded to oligosaccharides and then transported to the periplasm where it is further hydrolysed to monosaccharides which can then be transported to the cytoplasm of the cell. (B) Genomic content of *B. ovatus* ATCC 8483 PUL in its action on corn arabinoxylan.

Elsewhere Rogowski *et al.*^[35]^, 2015 showed that bifidobacterial growth on xylooligosaccharides (XOS) may be supported by *Bacteroides* species. Notably, *Bi. adolescentis *ATCC 15703 was unable to utilise xylans, although it can degrade simple XOS such as linear arabino-xylooligosaccharides. These authors showed that xylans can be divided into complex xylan, such as corn bran (CX), which contains several different linkages and distinct monosaccharides, or simple xylan, such as wheat arabinoxylan (WAX), which consists of a smaller number of different monosaccharides and linkages^[35]^. WAX was degraded by *B. ovatus *ATCC 8483 using two PULs [Figure 3A and B], and some of the resulting breakdown products were then further metabolised by *Bi. adolescentis *ATCC 15703. However, this glycan sharing was not observed with CX. The authors of this study suggested that *Bi. adolescentis *ATCC 15703 lacked the machinery to degrade the PBPs released by *B. ovatus *ATCC 8483 from CX due to the complexity of these oligosaccharides. Specifically, *B. ovatus* ATCC 8483 was shown to encode a GH98, which is located at the bacterial cell surface to start degradation of the CX backbone. The authors hypothesized that if the efficient machinery is introduced into *Bi. adolescentis *ATCC 15703, this bacterium will be able to cross-feed with *B. ovatus *ATCC 8483, even with complex xylans. To investigate this, they created a mutant strain of *B. ovatus *ATCC 8483 that lacked a functioning GH98 xylanase CAZyme (ΔGH98), thus preventing the cleavage of the backbone of CX and inhibiting its growth. When the ΔGH98 mutant was co-cultured with wild-type *B. ovatus *ATCC 8483 on CX media, growth of both strains was observed, which provides evidence that the wild-type strain releases PBPs from the breakdown of CX, which are then utilised by the mutant.

Further study into the utilisation of XOS by *Bacteroides *and *Bifidobacterium* strains in co-culture supported the findings of the previous study by Zeybek *et al.*^[36]^. Mono and co-culture fermentation experiments were carried out with various xylans as substrate; *Bi. bifidum*, *Bi. breve*, *Bi. longum* subspecies *infantis* and *Bi. longum* subspecies *longum* were unable to utilise XOS in monoculture; however, *Bifidobacterium animalis *subsp*. lactis* was able to grow on XOS but no other xylans. In monoculture, two* Bacteroides *strains, *B. ovatus *ATCC 8483 and* B. xylanisolvens *XB1A, showed good growth on xylose, XOS, beechwood xylan and corncob xylan. In co-culture, in the presence of the *Bacteroides *strains, *Bi. animalis *subsp. *lactis,* showed growth on beechwood and corncob xylans. *Bi. bifidum*, *Bi. breve*, *Bi. longum* subspecies *infantis* and *Bi. longum* subspecies *longum* showed no growth on any of the substrates in co-culture. These results highlight the differences in xylan-type polysaccharide fermentation abilities of different bifidobacterial species as is the case in the degradation of inulin-type fructans observed by Falony *et al.*^[37]^. The observed growth of *Bi. animalis *subsp. *lactis, *in co-culture, provides evidence for a cross feeding relationship between this species and the *Bacteroides.* This is likely due to the release of XOS as a PBP on the extracellular hydrolysis of xylan by the *Bacteroides* species. This released XOS is subsequently utilised by *Bi. animalis *subsp. *lactis. *This also provides an explanation for the lack of growth of other* Bifidobacterium* species in co-culture as they are unable to utilise XOS.

**Figure 4:**
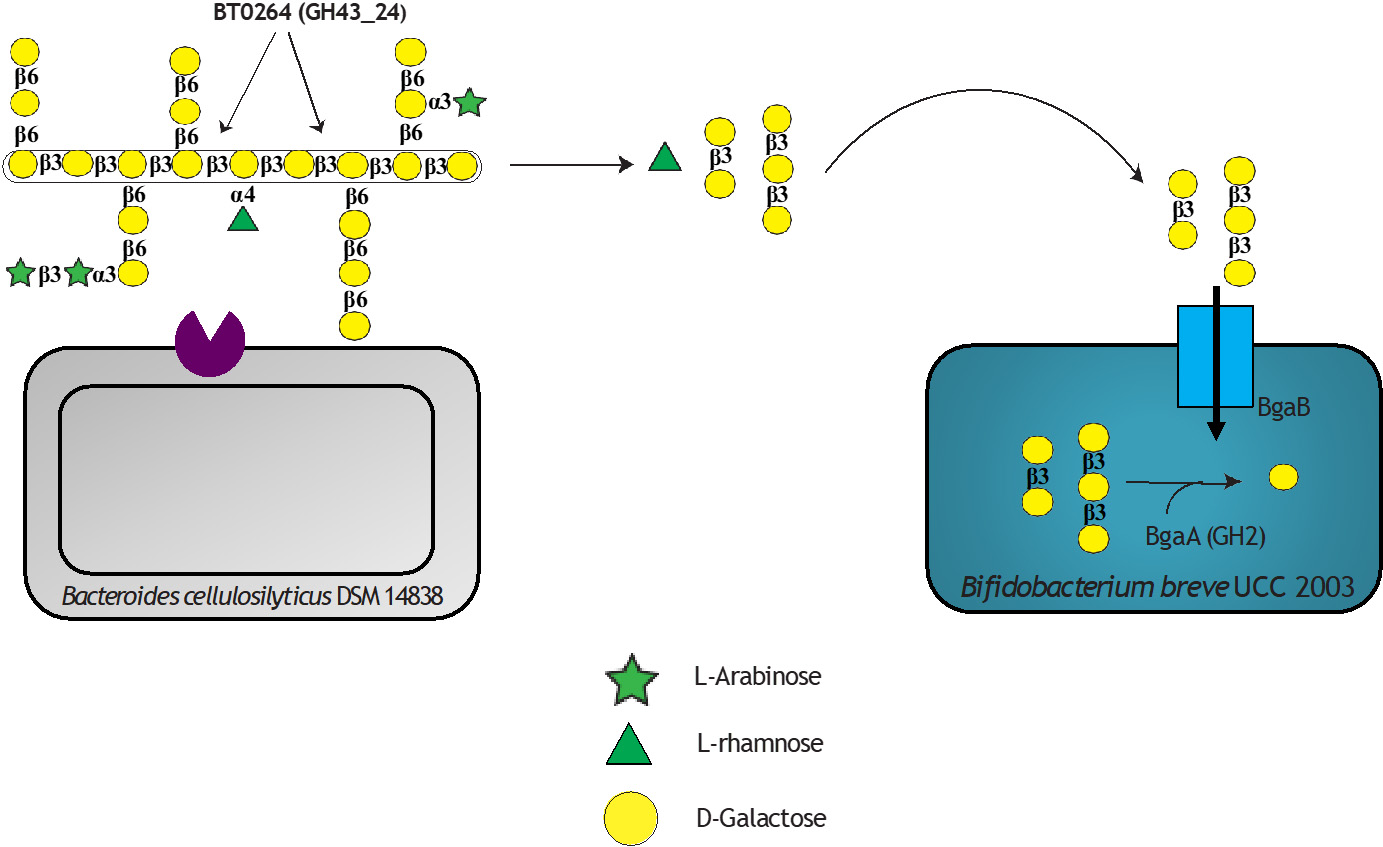
Molecular interactions between *B. cellulosilyticus *DSM 14838 PRL 2010 and *Bi. breve* UCC2003 when act on larch wood arabinogalactan. *B. cellulosilyticus *employs a GH43_24 on its surface to break down the complex polymer into smaller oligosaccharides releasing beta-1,3-galactobiose and beta-1,3-galactotriose and rhamnose into the medium. *Bi. breve* can use these galactooligosaccharides as carbon source degrading them to galactose using a specific GH2 in its cytoplasm. Galactose is later incorporated into the central catabolism of the cell.

Cross-feeding behaviour between *B. cellulosilyticus* DSM 14838 and certain bifidobacterial strains was analysed previously^[38]^, where interactions between the assessed strains were observed during cultivation on plant-derived Larch Wood arabinogalactan (LW-AG, Figure 4). This study demonstrated how *B. cellulosilyticus* DSM 14838 primarily degrades LW-AG to release rhamnose and β-1,3-galactooligosaccharides, the latter being further metabolised by certain bifidobacterial strains. *Bi. breve *UCC 2003 grown in co-culture with *B. cellulosilyticus *DSM 14838 was shown to grow on LW-AG as a carbon source, whereas no growth was detected in monoculture, thus revealing a cross-feeding activity between these two organisms. Further investigation showed that the carbohydrates β-1,3-galactobiose and β-1,3-galactotriose are utilised by *Bi. breve *UCC 2003. In addition to *Bi. breve *UCC 2003, also *Bi. longum *subsp. *infantis *ATCC 15697 was shown to utilise AG-derived oligosaccharides released by *B. cellulosilyticus *DSM 14838. The *bgaA* gene was identified in the genome of *Bi. breve *UCC 2003 and was predicted to encode a GH2 enzyme which is involved in the degradation of β-1,3-galactooligosaccharides. The active site of the BgaA enzyme was identified as being specific to β-1,3-galactobiose and β-1,3-galactotriose. Interestingly, this gene was not identified in other bifidobacterial species examined, including *Bi. breve *JCM 7017, *Bi. bifidum *LMG13195, and *Bi. longum *subsp. *longum *NCIMB8809, this being consistent with their inability to cross-feed with *B. cellulosilyticus *DSM14838.

Although no cross-feeding interactions were observed, a study by Liu *et al.*^[39]^ showed how certain *Bacteroides *and *Bifidobacterium *species are involved in the breakdown of polysaccharides in co-culture. Three polysaccharide mixtures, made up of different combinations of AX, xyloglucan, β-glucan and pectin, were used as the sole carbon source to perform co-cultivation experiments involving five different bacterial species, including *B. ovatus *ATCC 8483 and *Bi. longum *subsp. *longum *ATCC 15707. Size-exclusion chromatography showed that the observed degradation of the polysaccharides was carried out by *B. ovatus *ATCC 8483 and *Bi. longum* subsp. *longum *ATCC 15707, and the other bacterial species did not utilise these carbohydrates. Further analysis identified the release of oligosaccharides by *B. ovatus *ATCC 8483 on the hydrolysis of β-glucan; however, these glucooligosaccharides were not utilised by any of the other species in the co-culture. It was found that *B. ovatus *ATCC 8483 played a key role in the production of the SCFA succinate, which was utilised in the formation of propionate by other members of the co-culture, supporting the claim that *Bacteroides* species act as primary degraders of polysaccharides. This was also the case in the production of lactate by both *B. ovatus *ATCC 8483 and *Bi. longum *subsp. *longum *ATCC 15707, which was utilised by the other organisms. This study, along with previous studies discussed, highlights how the microorganisms in the human GIT can cooperatively interact in the breakdown of polysaccharides and benefit one another through PBPs and SCFA production as well as providing benefit to the human host.

## CONCLUSION AND FUTURE PERSPECTIVES

The abilities of various *Bacteroides* and *Bifidobacterium* species to utilise different polysaccharides have been highlighted in this review. The identification of polysaccharide utilisation loci in the dominant gut phyla, *Bacteroides*, has helped to increase knowledge of the complex system in which glycan molecules are broken down. It has also allowed for further research into these utilisation mechanisms as well as the ongoing characterisation of carbohydrate-active enzymes that are produced by all glycan-degrading bacteria, including *Bifidobacterium*. Understanding of enzyme function is essential to fully understand NDC utilisation and may help us predict the production of postbiotics, either SCFA or other bioactive compounds such as vitamins, from a given substrate. Enzymology also contributes to our understanding of the interactions between bacteria in the context of the complex community that is the human gut microbiota.

In the case of *Bacteroides* and *Bifidobacterium*, there is evidence for glycan-sharing and cross feeding activities between certain members of these two genera, particularly during the breakdown of dietary fibre. As has been discussed in this review, interactions between these two genera have previously been studied. However, it is likely that many other cross-feeding activities exist that have yet to be discovered. Interaction between *Bacteroides* and* Bifidobacterium* in the breakdown of arabinoxylan has been observed in a small number of studies; however, there is little to no literature available on possible cross-feeding activities on other dietary glycans, such as β-glucan, arabinogalactan, arabinan. Future research should investigate the interaction, if any, between *Bacteroides *and* Bifidobacterium *in the metabolism of dietary β-glucan and its effect on the human host. In addition, there are numerous other gut commensals, less dominant in the human large intestine such as *Lactobacillus reuteri*, which can be studied for their cross-feeding activities with *Bifidobacterium* (either involving dietary fibres or released mon-/oligo-saccharides), but also for the conversion of metabolic end products of one species (e.g., 1,2-propanediol, lactate) into other metabolites (propionate, butyrate). The experimental proof of the conservation of polysaccharide utilisation loci involved in β-glucan degradation specifically, amongst the *Bacteroides* would allow us to identify the enzymes involved in the metabolism of these molecules, which would then provide a starting point to investigate whether *Bifidobacterium *has a role in the breakdown of this polysaccharide. Due to the extremely complex nature of the human gut microbiota as well as the complexity and variation in carbohydrate structure, much more research is required for us to fully understand the roles of each of the members of the human gut microbiota.

## DECLARATIONS

### Copyright

© The Author(s) 2022.
